# A citizen science study of short physical activity breaks at school: improvements in cognition and wellbeing with self-paced activity

**DOI:** 10.1186/s12916-020-01539-4

**Published:** 2020-03-17

**Authors:** Josephine N. Booth, Ross A. Chesham, Naomi E. Brooks, Trish Gorely, Colin N. Moran

**Affiliations:** 1 Moray House School of Education and Sport, University of Edinburgh, Edinburgh, EH8 8AQ Scotland, UK; 2 Faculty of Health Sciences and Sport, University of Stirling, Stirling, FK9 4LA Scotland, UK; 3 School of Health, Social Care and Life Sciences, University of the Highlands and Islands, Centre for Health Sciences, Old Perth Road, Inverness, IV2 3JH UK

**Keywords:** Acute physical activity, Cognition, Children

## Abstract

**Background:**

School-based physical activity and running programmes, such as The Daily Mile™, are increasing in popularity globally. The aim of this research was to examine the acute impact of such classroom physical activity breaks on cognition and affective wellbeing.

**Methods:**

A total of 5463 school pupils from 332 schools took part in a citizen science project with a repeated measures design. They completed tasks of cognition (inhibition, verbal, and visuo-spatial working memory) and the Children’s Feeling Scale and Felt Arousal Scale before and after three different outdoor activities: a classroom break of 15 min of self-paced activity, a near maximal exhaustion activity (the bleep test), and a no-exercise control group where pupils sat or stood outside. Wellbeing and fitness were examined as mediators of the relationship between outdoor activity and cognition.

**Results:**

Fifteen minutes of self-paced outdoor activity was beneficial for pupils’ cognition and wellbeing in comparison to both other activities (Cohen’s *d* effect sizes ranging from 0.04 to 0.22; small). The relationship with cognition was not mediated by participants’ fitness level and was only partially mediated by wellbeing. Change scores for alertness were higher after the bleep test compared to the control activity but similar for all other outcomes.

**Conclusions:**

Taking a break from the classroom to complete 15 min of self-paced physical activity should be considered a worthwhile activity by class teachers, school management, and policymakers. Additionally, more intense physical activity should not be considered to be detrimental.

## Background

Physical activity (PA) is believed to have a beneficial impact on cognition and academic performance; however, the evidence for children and adolescents is inconsistent [1, 2]. For instance, a recent review of school-based activity found a 10–15-min bout of activity improved time-on-task, with higher intensities showing larger effects [3]. In contrast, 5 min of vigorous activity improved attention whereas longer bouts of moderate-to-vigorous physical activity (MVPA) did not [3]. Similarly, previous reviews have reported that PA *was* beneficial for working memory across the lifespan (e.g. [4]), whilst more recent reviews found no consistent impact in children [3]. Furthermore, a recent expert panel [2] highlighted the importance of adequate sample size, control groups, and research on the optimal duration and intensity of activity, as well as into underlying mechanisms by which PA may impact on cognition in children and adolescents.

Whilst there is some evidence of an impact of simple aerobic exercise on cognition, specifically executive function, more complex activities may be more beneficial [5]. The impact of PA on cognition may be mediated by improvements in mood and self-confidence and by reducing poor health and stress [5]. Indeed, Singh et al. [2] suggested that the relationship with psychosocial functioning may be one mechanism by which PA has an impact on cognition in children and adolescents. Additionally, there is evidence that taking part in PA outside can be more beneficial psychologically than the same activity completed inside [6]. Furthermore, there are inconsistencies in the literature concerning the role of fitness, with some reviews demonstrating positive associations with cognition in children [1] suggesting it is an important mediator, and others showing less consistent findings (e.g. [7]). Exploration of these potential mechanisms may lead to more consistency in the evidence base.

Despite inconsistencies in the literature, classroom PA breaks are being widely adopted (see [8]). Typically, these aim to increase activity and disrupt sedentary behaviour. The Daily Mile™ [9], where children run/walk outside for approximately 15 min each school day, is 1 example with growing popularity: now in over 10,000 schools across more than 77 countries [9]. It has been found to increase MVPA and reduce sedentary time [10, 11] and improve fitness and body composition [11]. Whilst the health benefits are no doubt a key consideration in implementation, classroom breaks take time away from academic lessons. The Daily Mile™ differs from other school-based running programmes, in that it occurs during class time and is designed as a genuine break from class activity, not an addition to existing break or lunch times [12]. Although reviews argue there is no long-term detriment to learning [2], the immediate impact of taking 15 min out of class in this manner (or 75 min a week) is not yet substantiated. This may be key information when teachers consider whether to devote time to PA breaks. A recent study reported that The Daily Mile™ had no immediate benefit for maths attainment or cognition in comparison to typical classroom activity [10]; however, this study did not control for key confounders (e.g. fitness) and so further research is needed.

Furthermore, the nature of The Daily Mile™, and perhaps a contributing factor to its success, is that the pace—running, walking, or a combination of both—is selected by the pupils [12]. Given that the optimum duration and intensity of activity required to impact on cognition remain unknown [2] and the inconsistencies in the evidence [3], it is important to determine how 15 min of self-selected activity like The Daily Mile™ compares to more intense bouts of movement. The present study addresses these significant gaps in the knowledge base.

### The present study

The present study, known as the Exercise Investigation, was a citizen science project in partnership with BBC Terrific Scientific [13]. Terrific Scientific investigations link mass participation of school pupils to research conducted by UK universities (e.g. [14]) and support children learning about science whilst producing valuable data. Teaching resources were supplied to schools, and teachers used their discretion to determine how to support pupils’ science learning (e.g. to align with topics/areas previously covered). Pupils collected their own data, and teachers were asked to discuss scientific methodology as part of this process. Resources (e.g. quizzes) were also prepared giving evidence-based information about physical activity and relationship with health, wellbeing, and the brain for use with pupils to increase their knowledge of the topic area.

At the end of data collection, pupils received a summary of their individual results and teachers received anonymised group-level results for their class to use for teaching purposes. Information given to teachers was designed to support discussion of physical activity and also the scientific methodology, including strengths and weaknesses of the design of the research and confounding factors so as to contribute to pupils understanding of science.

We aimed to examine the acute impact of classroom PA breaks on cognition by comparing a classroom break of 15 min of self-paced activity with a near maximal exhaustion activity (the bleep test) and a no-exercise control. This allowed determination of whether activity intensity influenced cognition and whether any impact was due to PA, or to taking an outside break from the classroom. Furthermore, as psychosocial health and aerobic fitness are potential mechanisms for this relationship, we examined the impact of activity on subjective wellbeing (affective component), and whether this and fitness had a relationship with cognition.

## Methods

### Participants and design

**Fig. 1:**
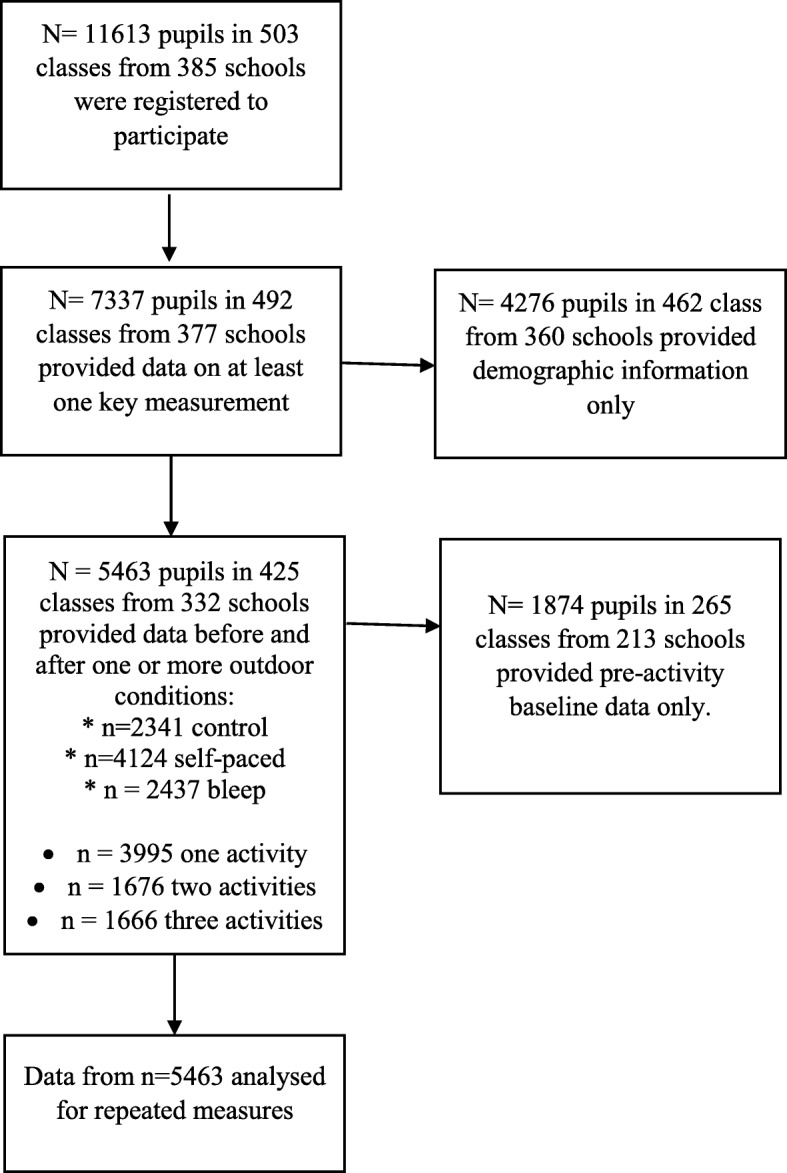
Participation and data provided

Participants were taking part in BBC Terrific Scientific. Recruitment was through BBC advertisement which included radio/TV advert, emails sent to schools, an information website, and social media campaign. Class teachers (*n* = 503) volunteered and registered their class for research. As this was an educational activity, pupils took part as they would any other class activity. They could choose not to take part in the research aspect though—i.e. not complete the outcome measurement. Table 1 shows demographic information. In total, 7337 children from 492 registered classes (mean age = 10.2 years, SD = 0.7; 50% female) provided information on at least 1 key outcome measurement after removal of outliers. Of that, 5463 pupils (74%; mean age = 9.7 years, SD = 0.64; 50.5% female) completed cognition and subjective wellbeing measurements before and after at least 1 of the outdoor activities (see Fig. 1). Participants came from all countries in the UK, although 36.6% of schools were in the least deprived areas (from IMD/SIMD areas 8, 9, and 10).

**Table 1:** Demographic information [*n* (%); mean (SD)]

	Male	Female	Total
Age (months)	122.34 (8.50)	122.29 (8.58)	122.31 (8.54)
Sex (*n*)	2702 (49.5%)	2761 (50.5%)	5463 (100%)
Country			
England	2082 (49.5%)	2124 (50.5%)	4206 (77.0%)
Scotland	394 (14.6%)	399 (14.5%)	793 (14.5%)
Northern Ireland	18 (0.7%)	19 (0.7%)	37 (0.7%)
Wales	201 (7.4%)	217 (7.9%)	418 (7.7%)
Crown Dependency	7 (0.3%)	2 (0.1%)	9 (0.2%)
School-level SES			
1 (most deprived)	165 (6.1%)	142 (5.1%)	307 (5.6%)
2	210 (7.8%)	192 (7.0%)	402 (7.4%)
3	175 (6.5%)	215 (7.8%)	390 (7.2%)
4	320 (11.9%)	292 (10.6%)	612 (11.2%)
5	232 (8.6%)	237 (8.6%)	469 (8.6%)
6	299 (11.1%)	313 (11.3%)	612 (11.2%)
7	336 (12.5%)	329 (11.9%)	665 (12.2%)
8	274 (10.2%)	283 (10.3%)	557 (10.2%)
9	359 (13.3%)	386 (14.0%)	745 (13.7%)
10 (least deprived)	325 (12.1%)	370 (13.4%)	695 (12.7%)
Age-corrected VO_2_ max (ml·kg^−1^·min^−1^)	48.45 (5.21)	46.68 (4.43)	47.55 (4.91)
Shuttle distance (m)	702.69 (402.08)	567.10 (331.17)	633.25 (373.61)

The data presented is from participants who provided data for repeated measures analysis, not the full sample who registered some of whom provided only baseline or demographic data

### Demographic information

Class teachers provided demographic information upon registration; this included school postcode to give an indication of socioeconomic (SES) grouping and pupil year group. Pupils were asked to specify their age and sex when completing initial measurements.

### Cognition

Cognition was measured using three computer-based tasks:

Inhibition was measured using an adapted stop-signal task [15]. Pupils were presented with a circle containing an arrow and asked to quickly press a computer button corresponding to the arrow direction, unless the stimuli changed colour, when they should supress their response (i.e. not press any button). Outcome variables were reaction time (for “go” trials), correct responses, incorrect responses (failure to stop), and an adjusted inhibition score (reaction time for go trials plus number of incorrect responses × 10) as recommended for reaction time tasks [16]. Using this method, lower scores equal better performance. The stop-signal task has acceptable reliability and validity in children [17], and the use of reaction times adjusted for error rate has been recommended for reaction time tasks [16].

Visual-spatial working memory was assessed using an adaption of the static boxes search task [18]. Pupils were presented with on-screen boxes to search for cartoon faces. The number of boxes and corresponding faces increased serially. Three rounds of boxes comprised one level with eight levels in total. Once a face was found, another would not be presented in that box until the next round. The task was adaptive: poor performance resulted in fewer levels being presented. Scores were based on accuracy with an optimum number of presses for the level reached (i.e. for level 4, optimum number of presses = 10) adjusted for the actual number of presses (actual–optimum) so that a lower score indicated better performance.

Verbal working memory was assessed using a reading span task [19]. Pupils were presented with a series of sentences and asked to judge their veracity before remembering the last word. The number of sentences presented together increased serially up to eight. A fuzzy logic algorithm accounted for spelling mistakes with 60% similarity denoting a sufficient match. Scores were based on the total number of words correctly recalled, with higher scores indicating better working memory. This method of scoring has good reliability in adults and children [20, 21].

### Subjective wellbeing

The adapted Children’s Feeling Scale and Felt Arousal Scale [22] assessed the affective component of subjective wellbeing. Children were presented with a Likert scale of facial expression pictures and asked: “How do you feel right now?” on a scale of very bad to very good (scored from − 5 to + 5) and “How awake do you feel right now?” on a scale of very sleepy to very awake (scored from 1 to 6). Following previous procedures [22], these items are analysed as two measures, and for both, a higher score indicates greater feelings of wellbeing. Single-item measures have acceptable validity [23], and this adapted version has been used widely with children (e.g. [24, 25]).

### Outdoor activities

Pupils were asked to complete all three different outdoor activities: (A) a self-paced run/walk activity (SPA), similar to The Daily Mile™, where the children ran or walked at a speed of their own choice for 15 min; (B) a 20-m bleep test (BPT) following standard procedure [26] (see below); and (C) a control activity (CON) where the children went outside to sit or stand for 15 min (ideally sit).

### Procedure

Ethical permission was granted from the University of Edinburgh ethics committee (UOE ref 1066). Information packs including health and safety information and information letters for parents were provided to schools. Following British Psychological Society ethical guidance, BBC Terrific Scientific was deemed an educational activity and parental opt-out consent was employed, in addition to school/teacher consent for class participation. Pupils who chose not to participate in the research did not complete the online tasks. Class teachers gathered this information.

Teachers completed an online form to register their class for BBC Terrific Scientific. Upon registration, access was given to a secure website containing the online tasks. Separate registration information was required from teachers to ensure that all information held by the research team was independent from the BBC. A set of unique pupil identifiers were computer generated for registered classes. Teachers allocated each consenting pupil in their class an identifier and retained this information throughout the project destroying it upon completion.

Consenting pupils were given access to the online system by their class teacher using the unique identifier. Pupils completed the demographic questions the first time they logged in and then tasks of cognition and subjective wellbeing before and after completing each outdoor activity. Class teachers selected the order in which the outdoor activities were completed and led the pupils in each activity. Teachers were instructed that (1) each activity (with associated pre- and post-measurements) should be on a different day; (2) this could be spread over 3+ days (e.g. 1 day per week); (3) pre-measurements should be completed immediately before the outdoor activity and should not be just after pupils arrived in the morning, just after break or lunch time, or just after completing PE (or other PA); and (4) post-measurements should be completed immediately (within 20 min) after the outdoor activity. Lesson plans, videos explaining the tasks and procedure, and downloadable pupil resources were given to class teachers to share with pupils prior to commencing the study (adapted copies accessible from [27]).

The BPT followed standard procedure [26]. A 20-m distance was marked using two parallel lines of cones or chalk. A bespoke audio file was created by the BBC to match the standard timing of bleeps and levels [28]. The test began at 8.5 km·h^−1^ and after each minute increased by 0.5 km·h^−1^. Pupils were grouped into pairs taking turns to act as runner or recorder. The runner ran in time with the bleeps whilst the recorder took note of each completed shuttle on recording sheets provided by the researchers. When the runner failed to reach the line by the bleep twice in a row, they were advised to stop. Pupils then swapped roles. Pupils then entered the level and shuttle number which they reached in the online form. Age-corrected VO_2_ max scores were created using procedures described by Léger et al. [26] as an indicator of participant fitness.

### Statistical analysis

Outliers were excluded using the interquartile rule (i.e. if they were < Q1 − 1.5 × IQR or > Q3 + 1.5 × IQR). Change scores associated with each outdoor activity were calculated for all outcome variables (post-outdoor activity–pre-outdoor activity). Differences in change scores between activities were examined using a linear mixed model with repeated measures and unstructured covariance. Residuals and probability plots were inspected to ensure assumptions were met. Confounders of age, sex, and SES were selected a priori and included in models along with all interaction terms. Cohen’s *d* effect sizes were calculated using estimated means and interpreted as small = 0.2, medium = 0.5, and large = 0.8. Pearson’s correlations were computed to examine the relationship between cognition, subjective wellbeing, and fitness. To explore the mediating impact of subjective wellbeing and fitness on the relationship between activity and cognition, bootstrapped estimates of the indirect effects and associated 95% confidence intervals (95% CI) were calculated using [29] and the MEMORE 2.0 macro for SPSS [30]. This approach is applicable for repeated measures designs with two time points [31]. Following this procedure, the difference between change scores associated with each outdoor activity is the outcome variable (*Y*) and the repeated measurement of the outcome and mediator (i.e. for each outdoor activity) becomes the predictor (*X* and *M* variables, respectively). To examine the mediating role of subjective wellbeing, both alertness and affect were entered simultaneously. Age-corrected VO_2_ max scores were treated as a constant in the model, rather than repeated measure, and so entered as a moderator variable. All analysis was performed using SPSS (v21).

## Results

Demographic information and descriptive statistics for outcome variables are presented in Tables 1 and 2. Change scores reveal positive trends for all outdoor activities.

**Table 2:** Descriptive information [mean (SD)]

Outcome	Self-paced activity			Bleep test			Control activity		
	Pre-test	Post-test	Change (*n* = 4124)	Pre-test	Post-test	Change (*n* = 2437)	Pre-test	Post-test	Change (*n* = 2341)
Affect	2.24 (1.99)	2.19 (2.70)	0.05 (2.75)	2.04 (2.41)	1.83 (2.83)	− 0.21 (3.07)	2.03 (2.36)	1.88 (2.67)	− 0.16 (2.62)
Alertness	4.31 (1.30)	4.51 (1.55)	0.22 (1.64)	4.35 (1.34)	4.35 (1.62)	0.04 (1.73)	4.21 (1.42)	4.10 (1.59)	− 0.10 (1.52)
Inhibition adj	803.43 (150.05)	767.47 (140.37)	− 36.46 (124.70)	772.93 (141.73)	766.15 (140.41)	− 1.72 (117.75)	777.65 (140.15)	763.40 (138.64)	− 8.29 (124.61)
Inhibition RT	648.95 (160.18)	649.38 (152.11)	− 4.74 (123.22)	640.72 (146.32)	651.62 (151.15)	8.77 (111.72)	651.90 (147.07)	650.51 (145.73)	2.63 (115.44)
Inhibition errors	12.02 (9.70)	9.06 (6.79)	− 2.47 (8.83)	10.26 (8.08)	9.08 (6.51)	− 0.74 (7.52)	9.68 (7.59)	9.01 (6.63)	− 0.51 (7.32)
Verbal WM	26.80 (15.22)	24.87 (15.73)	− 2.49 (13.61)	26.24 (16.01)	22.44 (15.54)	− 4.72 (13.84)	26.87 (15.75)	23.19 (16.41)	− 4.28 (13.27)
Visual-spatial WM	− 44.72 (37.21)	− 42.67 (37.77)	3.46 (34.98)	− 43.64 (37.73)	− 39.84 (36.51)	6.14 (34.25)	− 45.77 (38.01)	− 42.13 (37.56)	5.27 (34.61)

Sample size for SPA, bleep test, and control varied depending on outcome and whether pre- or post-test

*Inhibition adj* inhibition reaction time adjusted for errors, *Inhibition RT* mean inhibition reaction time for go trials, *Verbal WM* verbal working memory: total number of words, *Visual-spatial WM* visual-spatial working memory: actual adjusted for optimum

### Acute impact of outdoor activity on cognition

Differences in change scores after each outdoor activity were examined (Table 3). Activity was a significant predictor of all outcome variables in unadjusted models (overall model: alertness = *F* (2, 3127.28) = 33.94, *p* < 0.001, *d* = 0.12; affect = *F* (2, 3027.70) = 9.46, *p* < 0.001, *d* = 0.06; inhibition = *F* (2, 2983.56) = 66.16, *p* < 0.001, *d* = 0.17; verbal working memory = *F* (2, 2926.34) = 21.71, *p* < 0.001, *d* = 0.10; visual-spatial working memory = *F* (2, 3253.04) = 4.66, *p* = 0.009, *d* = 0.04).

**Table 3:** Results from analysis of change scores using linear mixed model: mean difference scores (SE), 95% CI, and effect sizes

Outcome	Self-paced activity vs. control				Bleep test vs. control				Self-paced vs. bleep test			
	Mean diff (SE)	95% CI	*p* value	Effect size	Mean diff (SE)	95% CI	*p* value	Effect size	Mean diff (SE)	95% CI	*p* value	Effect size
Affect												
Unadjusted	0.21 (0.07)	0.05 to 0.37	0.006	0.06	− 0.07 (0.08)	− 0.26 to 0.12	1.000	0.02	0.28 (0.07)	0.11 to 0.44	0.000	0.07
Fully adjusted	0.21 (0.07)	0.05 to 0.38	0.005	0.06	− 0.05 (0.08)	− 0.25 to 0.14	1.000	0.01	0.27 (0.07)	0.10 to 0.44	0.001	0.07
Alertness												
Unadjusted	0.32 (0.04)	0.22 to 0.41	0.000	0.15	0.13 (0.05)	0.02 to 0.23	0.014	0.06	0.19 (0.04)	0.10 to 0.28	0.000	0.08
Fully adjusted	0.31 (0.04)	0.22 to 0.41	0.000	0.15	0.13 (0.05)	0.02 to 0.23	0.017	0.05	0.19 (0.04)	0.09 to 0.28	0.000	0.09
Inhibition adj												
Unadjusted	− 28.03 (3.42)	− 36.22 to − 19.84	0.000	0.17	6.63 (3.67)	− 2.15 to 15.42	0.211	0.04	− 34.66 (3.25)	− 42.45 to − 26.88	0.000	0.22
Fully adjusted	− 27.93 (3.44)	− 36.16 to − 19.70	0.000	0.17	6.48 (3.70)	− 2.39 to 15.35	0.240	0.04	− 34.41 (3.29)	− 42.29 to − 26.54	0.000	0.22
Verbal WM												
Unadjusted	1.77 (0.36)	0.91 to 2.64	0.000	0.10	− 0.45 (0.40)	− 1.40 to 0.50	0.777	0.02	2.22 (0.37)	1.34 to 3.11	0.000	0.13
Fully adjusted	1.74 (0.36)	0.87 to 2.61	0.000	0.10	− 0.38 (0.40)	− 1.34 to 0.59	1.000	0.02	2.12 (0.38)	1.23 to 3.02	0.000	0.12
Visual-spatial WM												
Unadjusted	− 1.81 (0.94)	− 4.06 to 0.45	0.165	0.04	0.91 (1.01)	− 1.52 to 3.34	1.000	0.02	− 2.72 (0.92)	− 4.92 to − 0.51	0.010	0.06
Fully adjusted	− 1.89 (0.95)	− 4.15 to 0.38	0.140	0.04	0.62 (1.02)	− 1.84 to 3.07	1.000	0.01	− 2.50 (0.93)	− 4.73 to − 0.28	0.021	0.06

Fully adjusted models include age, sex, SES, and all interaction terms

*Inhibition adj* inhibition reaction time adjusted for errors, *Verbal WM* verbal working memory: total number of words, *Visual-spatial WM* visual-spatial working memory: actual adjusted for optimum

There was a statistically significant difference in change score associated with SPA compared to CON for all outcomes except the visual-spatial working memory task in both unadjusted and fully adjusted models (*p* values < 0.05); effect sizes were small (range 0.04 to 0.17). There were also statistically significant differences in all change scores when SPA was compared to BPT with effect sizes ranging from 0.06 to 0.22. There were no differences in change scores between BPT and CON for any outcomes, except alertness which was significantly lower after CON than after BPT. Furthermore, there were no statistically significant interactions with age, gender, or SES.

### Mediation analysis

There were statistically significant correlations between changes in alertness and affect and changes in verbal working memory associated with all activities (Table 4). Fitness (age-corrected VO_2_ max) was correlated with change score in alertness associated with SPA (*r* = 0.05, *p* < 0.05). This suggests that fitter pupils had greater increases in alertness after SPA, although the magnitude of the effect was small. There were no other statistically significant correlations, and so mediation was carried out for verbal working memory only.

**Table 4:** Correlation coefficients associated with each outcome and each outdoor activity

Outdoor activity	Outcome	Alertness		Inhibition adj		Verbal WM		Visual-spatial WM		Fitness	
		*r*	*p* value	*r*	*p* value	*r*	*p* value	*r*	*p* value	*r*	*p* value
Self-paced activity	Affect	0.57	0.000	− 0.01	0.595	0.07	0.000	0.02	0.324	0.03	0.201
	Alertness			0.01	0.727	0.05	0.008	0.01	0.796	0.05	0.041
	Inhibition adj					− 0.03	0.067	0.06	0.001	− 0.01	0.805
	Verbal WM							− 0.05	0.005	0.01	0.845
	Visual-spatial WM									− 0.03	0.199
Bleep test	Affect	0.53	0.000	− 0.03	0.229	0.10	0.000	− 0.03	0.223	0.04	0.060
	Alertness			− 0.02	0.332	0.05	0.026	0.03	0.165	0.01	0.675
	Inhibition adj					− 0.08	0.001	0.04	0.061	0.00	0.950
	Verbal WM							− 0.09	0.000	0.02	0.337
	Visual-spatial WM									− 0.01	0.706
Control activity	Affect	0.51	0.000	0.01	0.646	0.06	0.006	− 0.00	0.859	0.01	0.851
	Alertness			0.01	0.534	0.04	0.056	− 0.05	0.041	− 0.04	0.162
	Inhibition adj					− 0.07	0.002	0.04	0.077	0.01	0.674
	Verbal WM							− 0.05	0.022	0.05	0.083
	Visual-spatial WM									0.03	0.252

*Inhibition adj* inhibition reaction time adjusted for errors, *Verbal WM* verbal working memory: total number of words, *Visual-spatial WM* visual-spatial working memory: actual adjusted for optimum, *Fitness* age-corrected VO_2_ max

Mediation analysis (Table 5) shows that affect mediated the impact of SPA on verbal working memory compared to CON (*β* = − 0.11, SE = 0.06) and also when compared to BPT (*β* = 0.14, SE = 0.07). This was partial mediation only as the direct effect of SPA on verbal working memory remained significant. The direction of effects demonstrates that affect was greater after SPA when compared to CON and BPT, and that this positive relationship partially accounted for the improved performance on verbal working memory associated with SPA in comparison to CON and BPT. Alertness was not a significant mediator. There was no mediation in comparisons of CON with BPT.

**Table 5:** Mediation and moderation models for verbal working memory

	Total effect			Direct effect			Indirect effect			
	Beta (SE)	95% CI	*p* value	Beta (SE)	95% CI	*p* value	Beta (SE)	95% CI	Beta (SE)	95% CI
Control vs. self-paced	− 1.47 (0.01)	− 1.50 to − 1.44	0.000	− 1.43 (0.50)	− 2.42 to − 0.44	0.0046				
Affect							− 0.11 (0.06)	− 0.28 to − 0.02		
Alertness							0.07 (0.11)	− 0.14 to 0.30		
VO_2_ max → control									0.18 (0.08)	0.01 to 0.34
VO_2_ max → SPA									− 0.02 (0.09)	− 0.20 to 0.16
Control vs. bleep test	0.34 (0.02)	0.31 to 0.37	0.000	0.33 (0.52)	− 0.70 to 1.36	0.5271				
Affect							0.02 (0.03)	− 0.01 to 0.13		
Alertness							− 0.01 (0.05)	− 0.12 to 0.07		
VO_2_ max → control									0.10 (0.08)	− 0.06 to 0.26
VO_2_ max → bleep									0.06 (0.08)	− 0.10 to 0.23
Self-paced vs. bleep test	2.50 (0.01)	2.47 to 2.52	0.000	2.44 (0.51)	1.43 to 3.44	0.000				
Affect							0.14 (0.07)	0.04 to 0.31		
Alertness							− 0.08 (0.06)	− 0.23 to 0.02		
VO_2_ max → SPA									0.01 (0.08)	− 0.13 to 0.16
VO_2_ max → bleep									0.08 (0.08)	− 0.07 to 0.23

VO_2_ max = age-adjusted VO_2_ max and considered to be constant (e.g. not a repeated measure) therefore entered as a moderator in analysis

Age-adjusted VO_2_ max scores impacted change in verbal working memory scores associated with the CON, when compared to SPA only (Table 5). Participants who were fitter had greater improvements in verbal working memory after CON, but not with scores associated with SPA or BPT. *R*^2^ values in all cases indicated small effects with *R*^2^ = 0.005 (i.e. 0.5% of variance).

## Discussion

Taking part in a classroom break of 15 min self-paced outdoor activity was beneficial for pupils’ cognition and wellbeing compared to a bleep test (i.e. more intense activity), or a control activity of sitting/standing outdoors. The relationship with cognition was not impacted by participants’ fitness and was only partially mediated by wellbeing, so whilst pupils felt better after doing the activity, which was positively related to better cognitive performance, this was not the only reason performance on the cognitive tasks improved. For most outcomes, there was no difference between doing the bleep test or control, so whilst doing more intense PA was not as beneficial as a self-selected pace, it should not be considered detrimental. There was no interaction with age, gender, or SES in any of the analyses. Overall, this suggests an immediate positive impact of taking a classroom activity break of this nature.

### Relation to previous literature

Whilst there is evidence that PA is beneficial for cognitive performance and wellbeing, it is limited by small sample sizes, failure to consider mediating factors, and lack of consideration for activity intensity [2, 32, 33]. The present study goes some way to address these issues and provides evidence for the benefit of self-paced classroom PA breaks for cognitive ability and wellbeing in primary school pupils. Given the need to support school pupils to increase their PA levels [34], this may be a useful motivational tool for pupils, teachers, and parents.

In addition, we found that the bleep test (the most intense activity) was, in general, no different from the control activity of taking a break outside the classroom but was not as beneficial as the SPA. High Intensity Training (HIT) is known to be beneficial for cognitive function in children, although the impact differs between individuals [35]. Recent evidence in adults shows that preference for higher intensity activity is predictive of affective response [36]—i.e. those who feel better after intense PA are more likely to want to do intense activity than those who do not feel better. This individual variation supports the premise that a self-selected pace is preferable for both cognition and wellbeing.

Our findings differ from recent reports examining the acute impact of The Daily Mile™ in comparison to usual classroom activity [10]. Differing sample sizes, comparison groups, cognitive tasks, and confounding variables may be responsible for the varying findings. To examine cognition, we employed measures of inhibition, verbal, and visuo-spatial working memory. These executive functions are the underlying factors important for academic learning, and are vital support mechanisms for classroom behaviour. Indeed, improved executive function performance is predictive of long-term academic attainment, as well as health, wealth, and happiness (see [37] for a review). Whilst the effect sizes found in the present study are small, they are consistent with the magnitude of effects found for chronic PA interventions [33], longitudinal studies examining associations between PA and cognition (e.g. [38]), and other interventions to improve cognition [39]. Self-paced classroom PA breaks should therefore be considered a useful tool for supporting cognitive improvements in primary school aged children.

### Study strengths and limitations

The present study has several strengths and provides a unique contribution to our knowledge in this area. The large sample size and ability to consider mediating factors for the relationship between PA and cognition is novel and addresses many limitations of previous research. This citizen science project also contributes to pupils’ understanding of science more generally, as well as to the impact of PA. This approach to data collection does mean that fidelity of measurement for the bleep test is unclear, although the resulting figures for shuttle distance are very similar to those found in a sample of children when collected by trained researchers (e.g. [11]). It may also have impacted on the adherence to the instructions for the other outdoor activities, but would not have been an issue for other measurements due to the computerised assessment.

The nature of this research design (citizen science, teacher choice, and remote data collection) meant that we could not control the order in which pupils completed the PA tasks, or the exact content of what teachers said to pupils. Therefore, it is possible that order effects and teacher instruction may have influenced results, but we would expect that across this number of classes, these effects would be minimised. The order in which pupils completed the computerised tasks was randomised as part of the computer programme in order to control for possible order effects in the outcomes though. Pupil preferences for activities were not explored, and this would be an interesting addition to a further study.

The present study also examined pupils’ cognitive ability using tasks which are widely reported and well validated for this population and are more objective measures of cognitive skill than subjective reports of time-on-task [3]. Consistent with Lambourne and Tomporowski’s [40] findings for the measurement of acute effects, teachers were advised that cognitive tests should be completed as soon as possible after the end of the activity (no longer than 20 min post-activity); however, there was likely variation in this time interval which may have impacted the results.

We were not able to collect objective measurement of PA or sedentary behaviour from pupils and so cannot quantify how intense the activity was for each individual, or how much other PA pupils were undertaking (e.g. whether playing regular sport or actively commuting to school) nor whether the patterns of performance varied depending on habitual levels of PA. Whilst the inclusion of fitness data was a strength of our analysis, we recognise that the relationship between habitual PA and fitness is not straightforward and further research should aim to examine this more fully.

The evaluation of wellbeing and fitness as potential mechanisms for the relationship between PA and cognition is vital to further our understanding of this relationship and uncommon in this field [2]. However, we were not able to consider all possible mechanisms (e.g. sleep) or consider these classroom PA breaks in the context of movement across a 24-h period [41]. Further research should investigate these factors.

## Conclusions

Overall, we found that doing 15 min self-paced outdoor activity was more beneficial for school pupils’ wellbeing and cognitive performance in comparison to sitting/standing outdoors, or running to near exhaustion. Whilst doing more intense PA was not as beneficial as doing a self-selected pace, it was similar to control and should not be considered detrimental. The long-term health benefits of PA coupled with the acute cognitive benefits, which support learning, make such PA breaks worthwhile. These programmes are only one avenue by which young people can increase their activity levels and should be in addition to good quality PE and active transport where possible but should be considered by class teachers and school management, as well as policymakers.

## Data Availability

No data are available from this manuscript due to ethical and legal restrictions in place.

## Abbreviations

BPT: Bleep test
CI: Confidence interval
CON: Control activity
HIT: High Intensity Training
IMD/SIMD: Index of Multiple Deprivation/Scottish Index of Multiple Deprivation
MVPA: Moderate-to-vigorous physical activity
PA: Physical activity
PE: Physical education
SPA: Self-paced run/walk activity
SES: Socioeconomic status
WM: Working memory
